# The Impact of Challenge and Hindrance Stressors on Thriving at Work Double Mediation Based on Affect and Motivation

**DOI:** 10.3389/fpsyg.2021.613871

**Published:** 2021-01-28

**Authors:** Yi Yang, Xiang Li

**Affiliations:** Business School, Hunan Normal University, Changsha, China

**Keywords:** challenge stressors, hindrance stressors, thriving at work, self-efficacy, positive affect

## Abstract

Although the relationship between stressors and thriving at work has been established, the linkage between them is still in the early stages of theory development. This study proposed a two-path model, based on Lepine’s stressors-performance model, to analyze the effects of the stressors on the thriving at work. Two complementary mediating paths were proposed, i.e., affective strain (positive affect) and motivation (self-efficacy), which were explained using affective events theory and expectancy theory, respectively. Based on the empirical data from 233 employees, the results show that challenge stressors could enhance employees’ positive affect and self-efficacy, thus leading to thriving at work; on the contrary, hindrance stressors would result in negative influences. In addition, it is also found that the effect of affective path tend to be greater than that of motivation path, which could provide a practical guide for organizations to effectively apply stress management and to promote employees thriving at work.

## Introduction

Thriving at work is a mental state in which individuals experience both vitality and learning (Spreitzer, 2005), and it is closely related to employees’ attitudes, behavior, and performance (Porath et al., 2012; Walumbwa et al., 2017; Kleine et al., 2019). It has become one of the most researched conceptions in positive psychology (Porath et al., 2012), because increasing organizations expect employees not only to maintain positive affect in the workplace but also to learn and grow autonomously (Cameron et al., 2003). Although it has long been established (Flinchbaugh et al., 2015; Lin et al., 2020) that the stressors in the workplace, which are situational factors and have significant influences on the employees (Bliese et al., 2017), will impact thriving at work, the underlying mechanisms remain unclear, and the corresponding research is still in the early stages of theory development.

The mechanism of different stressors on outcome variables may be different (Podsakoff et al., 2007), making the work stress in a complex structure (Lazarus and Folkman, 1984). Some studies referred to work stressors as a negative factor that hinders thriving, and found that psychological stressors might cause negative affective, physiological and behavioral responses (Ganster and Rosen, 2013; Cullen et al., 2015). However, Prem et al. (2017) found that learning demand and time pressure significantly and positively predicted employees’ thriving; the empirical results of Flinchbaugh et al. (2015) showed that the existence of challenge stressors could improve life satisfaction through the intermediary role of thriving. When the work lacks pressure, some employees may slack at their work, which makes their performance decline (Rodell and Judge, 2009). There has not been a comprehensive and consistent conclusion on the relationship between stressors and thriving at work.

Moreover, most of the above studies only reported the superficial relationship between stress and thriving at work, while did not explore the underlying intermediate process of the relationship. Previous studies (Lepine et al., 2005; Rodell and Judge, 2009) have shown that motivation and affect are important mechanisms that link stressors and outcomes, such as work attitude. Workplace stressors, both challenge and hindrance, regarded as affective events, may generate affective responses, such as attentiveness, anger and anxiety. Such affective reactions will further related to workplace behaviors (Rodell and Judge, 2009). Podsakoff et al. (2007) explored the chain mediating variable between stressors and turnover behavior, and found that challenge stressors can positively affect employees’ work attitude (e.g., job satisfaction, organizational commitment, and turnover intention), thus reducing turnover behavior. In addition, a chain of evidence showed that both affect and work motivation are important antecedents of thriving at work. For example, Abid et al. (2020) proposed that prosocial motivation is an important intermediary between managerial coaching and thriving. All these works showed that there is not a simple direct relationship between stressors and thriving at work, but a complex mediating mechanism, and different types of stressors may have different effects on outcome variables (Podsakoff et al., 2007).

Cavanaugh et al. (2000) classified stressors in the workplace using the dichotomy of “challenge-hindrance” based on the good and bad attributes of stressors, which has proven to be a promising classification method by other scholars (O’Brien and Beehr, 2019) and helps organizations to improve the pertinence and effectiveness of workplace stress management, reducing operating costs and promoting performance improvement (Webster et al., 2010; Flinchbaugh et al., 2015; Webster and Adams, 2015). Inspired by this dichotomy classification and Lepine’s stressors-performance model, this work proposes a two-path model to investigate whether and how the challenge and hindrance stressors will influence thriving. The proposed model consists of an affective path and a motivation path, which are developed by introducing two mediating variables, i.e., positive affect and self-efficacy, from the perspective of “stressors-strain,” based on affective events theory (AET) and expectancy theory, respectively. The study reveals the “black-box” between stressors and thriving, which is conducive to fill the theoretical gap in this field and further promote the organizational practice of stress management and thriving at work.

## Literature Review and Research Hypothesis

### Thriving at Work

According to Spreitzer (2005), thriving at work refers to the psychological state that individuals experience both vitality and learning in their work at the same time. Spreitzer (2005) and Porath et al. (2012) developed and validated the construct of thriving at work, including “vitality” and “learning.” Vitality is the state of enthusiasm and motivation in employees (Nix et al., 1999), and learning is the ability of employees to improve their level and build self-confidence through knowledge acquisition (Carver, 1998). Although each dimension indicates a certain degree of personal growth and development in work, it is only when the two dimensions are combined and achieve a high level that they can promote each other and form the experience of thriving at work (Porath et al., 2012). Some empirical studies showed that the two-factor model of thriving at work has a higher data fit than other structures (Carmeli and Spreitzer, 2011; Niessen et al., 2012). Many studies showed that thriving at work can be used as a measure of personal growth perception, which helps people to understand what they are doing and how they are doing (Paterson et al., 2014; Spreitzer, 2005).

Existing studies have found that thriving at work is related to a wide range of positive effects on employees’ attitudes, behavior, and performance. Thriving is related to a series of (i) attitude variables, such as job satisfaction (Porath et al., 2012; Kleine et al., 2019), life satisfaction (Flinchbaugh et al., 2015), job engagement (Ren et al., 2015; Abid, 2016), organizational commitment (Kleine et al., 2019), career adaptability (Jiang, 2017), attitude toward self-development (Spreitzer, 2005; Paterson et al., 2014), less job strain and burnout (Porath et al., 2012); (ii) behavior variables, such as organizational citizenship behaviors (Porath et al., 2008), innovative work behavior (Wang T. et al., 2019; Wang Z. et al., 2019), voice behavior (Kim et al., 2020), taking-charge behavior (Zeng et al., 2020), less turnover intention and absenteeism (Abid, 2016); and (iii) some index variables, such as health (Walumbwa et al., 2017) and performance (Elahi et al., 2019). It also promotes organizational climate, performance (Spreitzer and Porath, 2013) and thriving at home (Porath et al., 2012).

Based on the socially embedded model of thriving at work (Spreitzer, 2005) and the integrative model of human growth at work (Spreitzer and Porath, 2013), the antecedent variables of thriving at work can be divided into following categories: (i) work context, such as perceived organizational support (Abid et al., 2015), information sharing, climate of trust (Kocak, 2016), leadership (Walumbwa et al., 2017) and stress (Flinchbaugh et al., 2015); (ii) work resource, such as knowledge (Jiang et al., 2019) positive meaning (Niessen et al., 2012) and positive affect (Taneva and Arnold, 2018); (iii) individual characteristics, such as proactive personality (Jiang, 2017), prosocial motivation and self-efficacy (Abid et al., 2020); (iv) agentic work behaviors, such as task focus (Niessen et al., 2012), exploration and learning (Paterson et al., 2014); and (v) basic psychological needs, including autonomy, competency and relatedness (Spreitzer and Porath, 2013).

### Stressors and Thriving at Work

Stressors are job-related factors that force an individual to deviate from his or her normal psychological or physiological functions (Beehr and Newman, 1978), which can be divided into two types: challenge stressors and hindrance stressors (Cavanaugh et al., 2000). The former refers to the work requirements that stimulate positive affect and promote personal growth by encouraging challenges to meet personal achievement needs, including workload, time urgency, work scope and responsibility, work complexity, etc. On the contrary, the latter refers to the work requirements that individuals think are too difficult to overcome, which will hinder the effective exertion of their abilities, produce negative affect, and affect career development, including organizational politics, role ambiguity, and conflict, bureaucratic procedures, job insecurity, etc. (Cavanaugh et al., 2000; Webster et al., 2010). Overall, different types of stressors have different effects on thriving at work.

Different types of stressors may have different effects on thriving at work. The socially embedded model of thriving at work illustrates the formation process of thriving and discusses how stable organizational context characteristics and dynamic resources jointly promote individual thriving at work. Individual agentic work behavior is the direct antecedent of thriving at work, which is caused by the characteristics of the work situation and the related resources (Spreitzer, 2005).

Challenge stressors stimulate employees’ thriving at work. Challenge stressors often mean that the organization puts forward higher work standards for employees and gives them a higher level of work authorization. When individuals are embedded in an atmosphere that encourages autonomous decision-making and when they enjoy discretion, wide information sharing, trust, and respect, motivational work behaviors are more likely to occur, thereby promoting thriving. This kind of work situation meets the basic psychological needs of employees, enabling them to have stronger learning motivation, and actively respond to challenges (Spreitzer and Porath, 2013). In addition, individuals will have a positive psychological expectation of challenging work requirements with challenge stressors (Webster et al., 2010). Simultaneously, this motivational behavior will lead to obtaining more work resources. Together with other resources provided by work, these positive psychological resources will promote individual motivational work behavior and further promote individual thriving. Therefore, once the organization offers the pressure of appropriate challenges, it can stimulate employees to form a closed loop between agentic behavior, work resources, and thriving at work, keeping employees in a state of continuous thriving.

On the contrary, hindrance stressors reduce employees’ thriving at work. For one thing, hindrance stressors symbolize a work situation with low autonomy and lack of trust and respect. Unreasonable arrangements and restrictions by organizations will reduce employees’ sense of belonging, make them unwilling to offer suggestions, and even lead to them consider quitting. For another, hindrance stressors will limit the production and application of work resources. Unreasonable organizational politics and bureaucracy make grass-roots employees unable to access the existing work resources of the organization, while role conflict means that employees do not have enough resources to complete multiple tasks at the same time (Schaubroeck et al., 2010). Therefore, with hindrance stressors, it is difficult for employees to experience thriving at work.

The above views are consistent with the findings of Lin et al. (2020), showing that different types of stressors play different mediating roles between transformational leadership and employees’ thriving at work. We therefore suggest the following hypothesis:

H1a: Challenge stressors are positively related to thriving at work.
H1b: Hindrance stressors are negatively related to thriving at work.

### Mediating Effect of Affect and Motivation

#### Lepine’s Stressors-Performance Model

Lepine et al. (2005) constructed a stressors-performance model based on cognitive interaction theory and expectancy theory, aiming to clarify the mechanical differences between the two types of stressors. They believed that affect and motivation play important roles between stressors and performance. The affective path is based on the cognitive interaction theory (Lazarus and Folkman, 1984). For an individual, the completion of challenge stressors has a specific promoting effect on personal growth, accompanying with potential benefits, which can stimulate employees’ positive affect and ability to adopt positive strategies, such as working harder. However, hindrance stressors are evaluated as pressures that hinder personal growth and have potential risks so that they can inhibit the generation of employees’ positive affect. They can even lead to adverse affects, avoidance behaviors, and extreme coping strategies, such as retreat and rationalization.

Expectancy theory explains why different types of stressors have different effects on job performance through motivation (Vroom, 1964). The relationship between the effort to meet job requirements, the probability of success (expectation) and the potential value or attractiveness (potency) may be related to the nature of stressors. In other words, the reason why challenge stressors can stimulate high work motivation is that people tend to think that there is a positive relationship between the effort required to deal with such job requirements (e.g., time urgency), the possibility of meeting such job requirements, and the valuable return after satisfaction. The reason why hindrance stressors can lead to low work motivation is that people do not believe that they can meet such job requirements (as in role conflicts), and they do not believe that meeting such job requirements can bring valuable returns. Therefore, Lepine et al. (2005) pointed out that challenge stressors and hindrance stressors can affect performance through affective strain (e.g., anxiety, depression, and frustration) and work motivation (e.g., effort, perseverance, sense of challenge, and learning motivation).

#### Mediating Effect of Positive Affect

Affect influences all aspects of people’s lives, such as people’s happiness, health and even life expectancy (Xu and Roberts, 2010). In the organizational context, researchers have observed that many variables influence employees’ attitude, behavior and job performance through affect. Positive affect, including joy, interest, content, love, and pride (Lazarus, 1991), is an individual’s unique immediate response to meaningful things and a temporary pleasure (Fredrickson, 1998; Fredrickson, 2001).

Affective events theory focuses on the structure, causes and results of affective reactions at work, and plays a unique role in explaining the “black-box” relationship between work environment and employees’ attitudes and behaviors (Ashkanasy et al., 2002). According to the theory, stable work environment can bring positive or negative work events, and affective experience of these events leads to individual affective reactions, which further influence individuals’ attitude and behavior (Weiss and Cropanzano, 1996). Stressors represent meaningful events because they provide information about the progress or hindrance of some valuable outcomes (Cavanaugh et al., 2000), and are the primary examples of affective events that may produce affective responses (Folkman, 2008). Based on the logic chain of AET, we focuses on the positive affective state generated by encountering stressors, because Fisher (2000) verified that work characteristics can well predict positive affective responses.

Challenge stressors can evoke positive affect because they are assessed as the opportunities for growth, learning, and goal achievement (Cavanaugh et al., 2000). Research showed that workload, time limit, work responsibility and complexity often bring employees positive affective reactions, such as happiness, hope and pride. These working environment characteristics can be identified as the source of challenge stressors (Wegge et al., 2006). From the individual’s view, challenge stressors contribute to increase income or accelerate growth, and compensate for the loss of individual resources so that it can stimulate individual’s positive affect. Lazarus (1991) believed that positive affect is a response to an encounter, indicating achievement and progress toward valuable results. For example, happiness is an affective feeling generated when the situation is evaluated as safe and familiar, or the event is understood as the progress and realization of personal goals. Pride is a positive experience when a goal is successful or evaluated as a success by others.

However, hindrance stressors negatively relate to employees’ positive affect because they are assessed as detrimental to personal growth and goal achievement. First, hindrance stressors, such as role ambiguity and role conflict, are mostly organizational problems, which are difficult to change through individual efforts, and are often evaluated as a troublesome or negative event, reducing individual positive affect. Second, this kind of pressure comes with very high personal psychological and physiological costs. Excessive cost consumption eventually can result in work burnout (Schaufeli et al., 2009), which involves affective exhaustion, a state of affective overuse, and extreme fatigue. We therefore suggest the following hypothesis:

H2a: Challenge stressors are positively related to positive affect.
H2b: Hindrance stressors are negatively related to positive affect.

After making clear that the event can produce affective response, AET further pointed out that the affective state stimulated by job requirements may lead to specific work attitude or behavior. The broaden-and-build theory of positive emotions indicated that positive affect cannot only expand the scope of individual’s momentary thought, bring immediate benefits to individuals, but also build lasting personal resources (e.g., intellectual resources, physiological resources, psychological resources, and social resources) and bring long-term adaptive benefits to individuals (Fredrickson, 2001).

Positive affect expands the individual’s mental activity space, develops individuals’ momentary thinking activity sequence, and encourages their thinking modes to be more active, open, and flexible. In the positive affective state, individual tendency can be boosted to seek diversity of learning (Fredrickson, 2013), and they can come up with more problem-solving strategies (Isen, 2004), which may promote their learning experience at work. For example, interest can generate a desire to explore and master new information and experiences, and to promote self-development in the process.

Moreover, positive affect can also construct individual psychological resources, such as resilience, optimism, a sense of identity, and goal orientation, which can prepare more favorable conditions for individual social adaptation and improve individual social adaptability. Positive affect had been found to be positively correlated with vitality (Wood et al., 1990; Ryan and Frederick, 1997), and Porath et al. (2012) defined vitality as a highly activated form of positive affect. In addition, a chain of evidence showed that there is a positive correlation between positive affect and thriving at work (Porath et al., 2012; Taneva and Arnold, 2018; Kleine et al., 2019). We therefore suggest the following hypothesis:

H3a: Challenge stressors promote thriving at work by stimulating individual positive affect.
H3b: Hindrance stressors inhibit thriving at work by weakening individual positive affect.

#### Mediating Effect of Self-Efficacy

According to expectancy theory, self-efficacy is a key factor in connecting work situation and individual cognition or behavior. The relationship between effort and success probability (expectation) may be related to the nature of stressors (Lepine et al., 2005). The characteristics of the work situation determine whether an individual has the ability to meet the job requirements, i.e., self-efficacy, which further determines personal attitude and effort (Vroom, 1964). For example, if a task both has all the necessary work resources, and the support of colleagues and leaders, the employee may judge that the task is more likely to be completed, so he is willing to work hard for it.

Self-efficacy is the degree of people’s self-confidence that they can use their skills to complete an assignment (Bandura, 1997). Bandura (1977) believed that there is also a kind of efficacy expectancy in addition to the result expectancy. Efficacy expectancy is people’s conjecture on their own behavior ability, which means whether people are sure that they can successfully carry out the behavior that brings certain results. When a person is convinced that he or she has the ability to carry out an activity, he or she may generate a high degree of self-efficacy and complete the assignment initiatively. For example, students will listen carefully when they know that concentrating on lectures can bring about ideal results and feel that they can understand what the teachers say. Self-efficacy is the basis of human activity. Some scholars of traditional achievement motivation theory regarded self-efficacy as a positive component of achievement motivation, and proposed to replace achievement motivation with self-efficacy as an explanatory factor of human behavior (Bandura, 1989). For example, Pintrich (2000) believed that learning motivation is mainly composed of value, expectation and affect.

Challenge stressors contribute to improving self-efficacy. Social persuasion is an important external factor influencing individual self-efficacy, referring to encouragement, trust, praise or reward from others (such as leaders), which confirms that individuals have the ability to complete tasks. People are more likely to have positive beliefs in their abilities when they are persuaded that they are capable of accomplishing tasks (Bandura, 1997). When employees are requested by the organization to complete more challenging tasks in a short period of time, they are often perceived as “a kind of expectation and recognition of their abilities.” The organizational trust helps to enhance their self-efficacy (Prem et al., 2017). In other words, challenge stressors can stimulate high self-efficacy because people tend to assume that there is a positive relationship between the effort to deal with such requirements (e.g., time urgency) and the possibility of meeting such requirements.

On the contrary, hindrance stressors reduce self-efficacy. Employees may feel helpless and fall into self-doubt due to the fuzziness and uncontrollability of assignments (Dickerson and Kemeny, 2004). When people’s work characteristics will not likely give them normal returns or the difficulty of work exceeds the regular requirements, it is difficult for them to maintain a good psychological state (Hackman and Oldham, 1976). For example, when employees experience role conflict situations, they are difficult to complete the conflicting tasks simultaneously. When employees no longer believe that their efforts can improve their working environment and outcomes, they may feel helpless, lack of self-confidence, which may inhibit their self-efficacy (Dickerson and Kemeny, 2004). In addition, different working environments provide people with different information (Bandura, 1989). When individual enters a strange and easily anxious situation, self-efficacy is easy to reduce; on the contrary, if individual enters a creative and cooperative organization, self-efficacy is easy to improve (Ding and Li, 2016). We therefore suggest the following hypothesis:

H4a: Challenge stressors are positively related to self-efficacy.
H4b: Hindrance stressors are negatively related to self-efficacy.

Self-efficacy is positively correlated with thriving at work. Expectancy theory points out that the degree of individual effort depends on the probability analysis of the realization of personal goals. Employees with high self-efficacy generally have a positive psychological prediction of the future believing that work can bring positive results to themselves, so as to focus more on tasks and establish close ties with other colleagues (Wu, 2001). These agentic behaviors are the engine of thriving at work (Spreitzer, 2005). Also, when the self-efficacy of employees is improved, they tend to adopt positive coping strategies (Chang et al., 2010; Ben and Auton, 2014). They regard challenges and pressures at work as good opportunities to learn new skills. They do not shrink back or give up in the face of difficulties but work harder to learn relevant knowledge and skills, which effectively alleviates the negative impact of challenge stressor on themselves (Stumpf et al., 1987). The self-determination theory also supports this view. When a task makes employees feel competent, the resulting self-efficacy will enhance employees’ internal motivation, increase their interest in work, and encourage spontaneous action, so that employees can thrive at work (Deci and Ryan, 2008).

Some empirical results also supported the positive correlation between self-efficacy and thriving at work. For example, Paterson et al. (2014) found that psychological capital (of which self-efficacy is an important component) has a strong role in promoting employees’ thriving; Zhu et al. (2018) found that people who are more confident in their own abilities are easier to experience thriving.

Combined with previous views, we also predict that self-efficacy is an important mechanism to link stressors with thriving. Although there is no direct research on the mediating role of self-efficacy between stressors and thriving, some empirical studies supported this conclusion. Jex and Bliese (1999) found that self-efficacy is a mediator between time pressure, workload, task characteristics and physical and mental stress. In addition, Abid et al. (2020) found that self-efficacy plays a mediating role between managerial coaching and thriving at work. We therefore suggest the following hypothesis:

H5a: Challenge stressors promote thriving at work by enhancing self-efficacy.
H5b: Hindrance stressors inhibit thriving at work by reducing self-efficacy.

**FIGURE 1:**
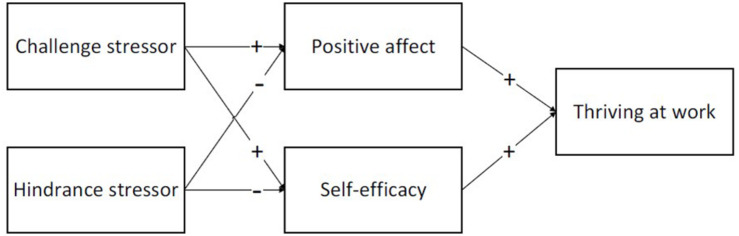
Research model.

Based on the above analysis, this paper proposes a research model, as shown in Figure 1. Challenge and hindrance stressors work together through two mediating pathways: affective strain (positive affect) and motivation (self-efficacy).

## Research Methods

### Sample and Procedure

We sampled companies from different industries to ensure that there is enough variation in individual’s thriving at work. 15 companies from the Pearl River Delta region of China participated in the survey, involving Internet, real estate, machinery manufacturing, finance, and other fields. The respondents were ordinary employees of enterprises. These companies are different in size, year of establishment, industry, form of ownership, and geographical location. Our sampling plan is to have at least 20 employees from each company participating voluntarily and anonymously in the questionnaire survey. We sent a link of an online questionnaire to the participants through the HR manager and provided a confidentiality commitment.

In order to reduce the influence of homologous method bias, we adopted the following control methods: (1) participants anonymously adjusted the questionnaire; (2) balanced the order of items to reduce participants’ guessing about the purpose of measurement; (3) conducted a pre-test before the formal survey to improve the scale items.

Finally, 223 valid questionnaires were obtained. The average age of the respondents was 29.5 (*SD* = 0.493), and the average length of service was 7.06 years (*SD* = 2.46). Among all the samples, 59% were female, 52% were married, and 80.7% received bachelor degree or above. The specific results are shown in Table 1.

**TABLE 1:** Sample characteristics.

**Variables**		**Frequency**	**Percent**
Gender	Female	132	49%
	Male	92	41%
Age	Below 25 years	50	22.4%
	25—35 years	142	63.7%
	Above 35 years	31	13.9%
Education level	College degree or below	43	19.3%
	Bachelor’s degree	102	45.7%
	Master’s degree or above	78	35%
Fertility status	Childless	135	60.5%
	1 child	64	28.7%
	More than 1 child	24	10.8%
Marital status	Married	116	52%
	Unmarried	107	48%
Working tenure	0–5 years	54	24.2%
	6–10 years	157	70.4%
	Above 10 years	12	5.4%

### Measures

We use the mature scale and ensure the accuracy of the scale through translation-back translation. First of all, two graduate students of business management major translated the scale into Chinese, and then one English major graduate student translated the Chinese scale back into English. After repeated discussions, they reached a consensus and finally formed the Chinese version of the scale. Before the formal distribution of the questionnaire, 5 MBA students were asked to fill in the questionnaire. We corrected the inappropriate expression according to the doubts.

The questionnaire consists of four subscales: challenge-hindrance stressor scale, self-efficacy scale, positive affect scale, and thriving at work scale. There are two dimensions in the challenge-hindrance stressor scale and the thriving at work scale. The four subscales were designed according to the existing literature to ensure the reliability and validity of the scale. The above scales all use a Likert five-point scale, where 1 represents “very inconsistent” and 5 represents “very consistent.”

Challenge-hindrance stressors. According to Rodell and Judge (2009), the stressors scale was divided into two dimensions: challenge and hindrance, and each dimension had four themes. For example, “Recently, I have to work very hard to finish my work” and “Recently, I have to go through a lot of red tape to finish my work.” The Cronbach α of the two dimensions were 0.803 and 0.809, respectively.

Self-efficacy. The dimensions of self-efficacy in the psychological empowerment scale compiled by Spreitzer (1995) and revised by Li et al. (2006), according to the Chinese situation, included three items, for example, “No matter what changes in the organization, I believe I can handle it well.” The Cronbach α was 0.875.

Positive affect. The Warr (1990) positive affect scale in work was used, comprising six items, “relaxed, satisfied, calm, optimistic, enthusiastic, and happy.” The Cronbach α was 0.802.

Thriving at work. The thriving at work scale developed by Spreitzer and Porath (2013) is divided into two dimensions. The learning scale includes five statements such as “I am active in learning,” and the vitality scale also includes five statements such as “I feel energetic.” The overall Cronbach α was 0.899.

Control variables. In order to determine the potential impact of other explanations on the empirical results, after a thorough review of the empirical studies on thriving at work, we chose age, gender and education level as control variables, which were also adopted by many scholars with thriving or stressors research (Li et al., 2019; Abid et al., 2020; Lin et al., 2020). Firstly, age is crucial to thriving, since elder people are more likely to be tired at work and unwilling to learn new knowledge (Kanfer and Ackerman, 2004). Secondly, Purvanova and Muros (2010) has proved that women tend to be more exhausted and feel less vital than men, which is an important component of thriving. Therefore, gender was set as a control variable. Finally, we choose education level as the control variable, because there are significant differences in perceived stress among employees with different educational levels (Golubic et al., 2010).

### Statistical Analysis

First, SPSS software was used to test the common method deviation. The mean value, standard deviation, correlation, and measurement reliability of each variable were statistically analyzed to provide the necessary support for the subsequent structural equation model test. Second, M-plus software was used for confirmatory factor analysis. Third, since there are different mediating paths in this study, M-plus was used to construct and test the dual mediation model. Last, the Monte Carlo method was used to test the mediating effect in the hypothesis model.

## Data Analysis

### Common Method Variance Analysis

In this paper, the Harman single-factor method was used to verify the common method deviation problem (Podsakoff and Organ, 1986). The results of principal component analysis without rotation showed that the common method deviation was not significant. This is because the eigenvalues of six factors were greater than 1, and the variance explained by the first factor was only 33.267%, which was lower than the critical standard of 40%.

### Descriptive Statistics and Correlation Analysis of Variables

Table 2 shows the average standard deviation and Pearson correlation coefficient matrix of five variables: challenge stressors, hindrance stressors, thriving at work, self-efficacy, and positive affect. Challenge stressors are significantly positively correlated with self-efficacy (*r* = 0.326^∗∗^), positive affect (*r* = 0.322^∗∗^), and thriving at work (*r* = 0.392^∗∗^). Hindrance stressors are negatively correlated with self-efficacy (*r* = −0.177^∗∗^), positive affect (*r* = −0.260^∗∗^), and thriving at work (*r* = −0.286^∗∗^). Self-efficacy (*r* = 0.548^∗∗^) and positive affect (*r* = 0.615^∗∗^) are significantly positively correlated with thriving at work. These correlations preliminarily verify hypotheses H1a-b, H2a-b, H4a-b, and provide necessary data support for the subsequent model test.

**TABLE 2:** Descriptive statistical results of variables and correlation coefficient matrix.

**Variables**	***M***	***SD***	**1**	**2**	**3**	**4**	**5**	**6**	**7**	**8**
(1) Gender	0.41	0.49	1							
(2) Age	29.54	6.99	0.081	1						
(3) Education level	3.17	0.90	0.029	0.094	1					
(4) Challenge stressor	3.49	0.72	0.231**	0.016	0.114	1				
(5) Hindrance stressor	2.71	0.84	0.107	−0.068	0.017	0.152*	1			
(6) Self-efficacy	3.95	0.70	0.116	0.105	0.022	0.326**	−0.177**	1		
(7) Positive affect	3.38	0.68	0.233**	0.073	0.053	0.320**	−0.260**	0.461**	1	
(8) Thriving at work	3.55	0.66	0.219**	0.059	0.151*	0.392**	−0.286**	0.548**	0.615**	1

M is the mean value, SD is the standard deviation, **p < 0.01, *p < 0.05.

*Gender: male = 1, female = 0; Education level: below college degree = 1, college degree = 2, bachelor’s degree = 3, master’s degree = 4, doctor’s degree or above = 5.*

### Confirmatory Factor Analysis

**FIGURE 2:**
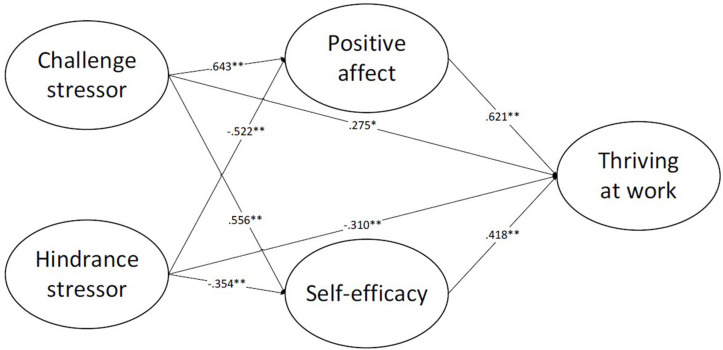
The relationship model of challenging and hindrance stressors, self-efficacy, positive affect, and thriving at work.

M-plus software was used to analyze the discriminant validity of the five key variables. Five-factor, four-factor, three-factor, two-factor, and single-factor models were compared, respectively (Table 3). Table 3 shows that the five-factor model data fit best, indicating that the five variables involved have good discriminant validity and are significantly better than other factor models. It shows that these five variables can represent five different constructs, and the next step of the data analysis can be carried out. Finally, the path coefficient diagram shown in Figure 2 is obtained.

**TABLE 3:** Results of confirmatory factor analysis of measurement model.

**Measurement model**	**χ^2^**	***df***	**χ^2^/*df***	**RMSEA**	**CFI**	**TLI**
Five-factor model (*X1, X2, M1, M2, Y*)	851.226	390	2.18	0.073	0.854	0.838
Four-factor model (*X1, X2, M1* + *M2, Y*)	1058.187	393	2.69	0.087	0.789	0.768
Three-factor model (*X1* + *X2, M1* + *M2, Y*)	1366.979	396	3.45	0.105	0.692	0.664
Two-factor model (*X1* + *X2* + *M1* + *M2, Y*)	1544.255	399	3.87	0.114	0.636	0.605
One-factor model (*X1* + *X2* + *M1* + *M2* + *Y*)	1635.501	402	4.07	0.117	0.609	0.577

*X1 = challenge stressor, X2 = hindrance stressor, M1 = positive affect, M2 = self-efficacy, Y = thriving at work.*

### Double Mediation Model Test

Considering all the one-way paths, a dual mediating model with positive affect and self-efficacy as mediating variables was established for path analysis. The final results are shown in Figure 2. The fitting indexes are: χ^2^/*df* = 2.18; RMSEA = 0.073; CFI = 0.854; TLI = 0.838. The model has a good fit, and the hypothesis model was established. Challenge stressors are directly and positively related to thriving at work (β = 0.275, *p* < 0.05), while hindrance stressors are directly and negatively related to thriving at work (β = −0.310, *p* < 0.01). Therefore, hypothesis 1a and 1b is empirically supported. Moreover, challenge stressors are positively related to positive affect (β = 0.643, *p* < 0.01) and self-efficacy (β = 0.556, *p* < 0.01), while hindrance stressors are negatively related to positive affect (β = −0.522, *p* < 0.01) and self-efficacy (β = −0.354, *p* < 0.01). At the same time, positive affect (β = 0.621, *p* < 0.01) and self-efficacy (β = 0.418, *p* < 0.01) can promote thriving at work, respectively. These results provide basic support for hypothesis 2a-b and 4a-b, suggesting that positive affect (as an affective mediator) and self-efficacy (as a motivational mediator) may play an indirect mediating role between stress and thriving.

### Bootstrap Test for Mediating Effect

We used the bias-corrected bootstrap method to analyze the difference in the double mediating effect. One thousand bootstrap samples were randomly selected. The estimated value of the mediating effect was obtained according to the samples, and the average path value of the mediating effect was calculated. If the confidence interval includes 0, the mediating effect or path coefficient is not significant (Wen and Ye, 2014). Table 4 shows that positive affect (0.203, 0.595) and self-efficacy (0.107, 0.358) have significant positive mediating effects between challenge stressors and thriving at work. On the contrary, positive affect (−0.495, −0.153) and self-efficacy (−0.224, −0.053) have significant negative mediating effects. Therefore, hypotheses 3a-b and 5a-b are verified.

**TABLE 4:** Test results of specific mediating effect based on Bootstrapping.

**Route**		**Effect value**	**95% confidence interval**		**VAF**
			**Lower limit**	**Higher limit**	
**Direct effect**	Challenge stressor → thriving at work	0.275^sig^	0.009	0.542	30.4%
	Hindrance stressor → thriving at work	−0.310^sig^	−0.546	−0.075	39.7%
**Indirect effect**	M1:challenge stressor → positive affect → thriving at work	0.399^sig^	0.203	0.595	44%
	M2: challenge stressor → self-efficacy → thriving at work	0.232^sig^	0.107	0.358	25.6%
	Total indirect effect	0.631^sig^	0.310	0.869	69.6%
	M1–M2	0.167^sig^	0.096	0.237	
	M3: hindrance stressor → positive affect → thriving at work	−0.324^sig^	−0.495	−0.153	41.4%
	M4: hindrance stressor → self-efficacy → thriving at work	−0.148^sig^	−0.244	−0.053	18.9%
	Total indirect effect	−0.472^sig^	−0.674	−0.271	60.3%
	M3–M4	−0.251^sig^	−0.251	−0.100	

*sig, significant; VAF, variance accounted for.*

Considering the difference of the two mediating paths’ effect value, we tested whether affect has a stronger mediator effect than self-efficacy. We evaluated the statistical difference (M1–M2: 0.167^sig^, M3-M4: −0.251^sig^), which shows a significant difference between both indirect effects.

## Discussion

With the intensification of enterprise competition and the acceleration of work rhythm, people are generally facing a lot of pressure from the workplace. At the same time, with the improvement of the complexity and professionalism of organizational work, employees are required to be more energetic and constantly learn and grow (Cameron et al., 2003). Stressors and thriving at work have been paid increasing attention in the field of organization and management, respectively, and have become an essential aspect of enterprise management. Although previous studies have linked stressors with thriving at work, the results are not consistent and rarely explain the underlying mechanism of the relationship. Referring to the stressors-performance model proposed by Lepine et al. (2005), based on the AET of Weiss and Cropanzano (1996) and the expectancy theory of Vroom (1964), this paper constructs and tests a dual-path model based on affect and motivation to explain the relationship between challenge and hindrance stressors and thriving at work.

### Theoretical Significance

The study verified the difference in the impact of different stressors on thriving at work. In terms of the overall effect, challenge stressors promote thriving at work, while hindrance stressors inhibit thriving at work, which was supported by our results. There may be essential differences in the nature of different stressors (Cavanaugh et al., 2000). “Good” stressors are related to high thriving at work, while “bad” stressors are related to low thriving at work. The results also indirectly support the existing research conclusions, that is, challenge stressors are related to high job performance, and hindrance stressors are related to low job performance (Pearsall et al., 2009), because thriving is the proximal antecedent of job performance (Elahi et al., 2019).

Although stressors have a significant direct impact on thriving at work, our study finds that stressors also have a significant indirect impact on thriving at work through affective strain and work motivation, thus uncovering the relationship between stressors and thriving. Stressors stimulate employees’ work affect and induce their work motivation, which leads to their thriving at work. This study focuses on the mediating role of employees’ affective state when they encounter stressors. In our model, challenge stressors have a positive indirect impact on thriving by stimulating positive affect, while hindrance stressors negatively impact thriving by weakening positive affect. These results show that the “good or bad” degree of stressors depends on whether the stressors can make employees feel happy. We also note that some studies showed that challenge stressors, such as time stress, may lead to more negative affect, such as anxiety and anger (Rodell and Judge, 2009). Although challenge stressors may counteract indirect effects on thriving at work through positive and negative affect, on the whole, positive affect is indeed an important mediating mechanism between stressors and thriving at work.

In the complementary mediator model, we found that the mediating effect of positive affect (M1 = 0.399^sig^, M3 = −0.324^sig^) is stronger than that of self-efficacy (M2 = 0.232^sig^, M4 = −0.148^sig^). In other words, our results mean that stressors may affect thriving at work more through affective path. Although there is no literature directly comparing the mediating effects of positive affect and self-efficacy between stressors and thriving at work, some related studies indirectly support our viewpoint. For example, the meta-analysis results of Lepine et al. (2005) showed that the estimated true correlation (*r*_c_ = 0.40) between affective strain and job performance is greater than that between work motivation and job performance (*r*_c_ = 0.16), and Porath et al. (2012) drew the same conclusion. Their data showed that the correlation coefficient between positive affect and thriving at work (*r* = 0.52) is greater than that between core self-evaluation (closely related to self-efficacy) and thriving (*r* = 0.46).

The results of the study are consistent with the logic of the broaden-and-build theory of positive emotions. Thriving at work is a mental state in which individuals experience both vitality and learning (Spreitzer, 2005). Vitality is a state of enthusiasm and motivation in employees (Nix et al., 1999), and learning is a kind of cognitive experience, which is a feeling that individuals are acquiring and can apply knowledge and skills to work (Elliott and Dweck, 1988). The broaden-and-build theory of positive affect believes that positive effect can not only expand the scope of individual’s momentary thought, bring immediate benefits to individuals, but also build lasting personal resources (e.g., intellectual resources, physiological resources, and psychological resources) and bring long-term adaptive benefits to individuals (Fredrickson, 2001). In other words, a positive effect not only makes individual’s thinking mode unusual, flexible and creative immediately, but also has been constructing the individual’s intellectual and psychological resources for a long time, promotes the individuals to acquire new knowledge and develop the ability to solve problems, to make employees’ vitality and learning at a high level at the same time, and achieves a thriving state (Spreitzer, 2005). Therefore, for thriving at work, positive affect is a more direct influence factor.

Self-efficacy is generally produced after the cognitive judgment of job requirements and self-ability (Bandura, 1997). There is a certain degree of delay in influencing stressors on self-efficacy and self-efficacy on thriving at work. In addition, self-efficacy is often realized through the mediating process of choice, thought, motivation, and psychosomatic reaction (Bandura, 1997). For example, the latest research of Abid et al. (2020) shows that self-efficacy affects thriving at work through prosocial motivation. Therefore, compared with positive affect, the impact of self-efficacy on thriving is less immediate.

### Management Implications

The results show that different types of stressors have different effects on employees’ job flourishing, indicating that managers should pay more attention to the effective management of employees’ stress. An indiscriminate reduction in work pressure won’t likely improve the learning enthusiasm or work vitality of employees. Therefore, managers need to distinguish the internal stressors of the organization effectively and improve the employees’ thriving at work by changing the hindrance stressors (such as role ambiguity, organizational politics, and other phenomena) and implementing the challenge stressors (such as increasing the workload and difficulty of work appropriately). Only by comprehensively preventing the generation of work pressure, which is difficult for employees to change through their own efforts, can we provide an independent and respectful platform for employees. In such working atmosphere, employees can seize the challenging job opportunities and realize their own growth and development.

Managers should adjust employees’ work motivation and affect so that employees can achieve a prosperous working state. In particular, they should attend to the stimulation of employees’ positive affect due to their more evident stimulating effect on thriving at work. Thriving at work provides a detection tool for employees’ perception of self-development and progress. It functions as a thermometer, helping employees read their psychological state immediately, judge whether it is overheated (likely to cause burnout) or too cold (likely to block development), and make timely adjustments.

For one thing, because short-term work events easily stimulate affect, negative work events such as role conflict should be avoided in the short term. Employees could be given appropriate work authorization to limit the work hours and tasks to be completed. Appropriate stress events can stimulate employees’ positive affect in a short period of time. They can improve concentration and enable more efficient completion of tasks. In addition, managers can also directly stimulate employees’ positive affect by organizing outdoor activities and giving rewards.

For another, managers should pay attention to the characteristics of the working environment. As far as possible, they should avoid organizational politics and bureaucracy. They should aim to create an organizational atmosphere consisting of leaders’ support and colleagues’ trust. Organizational politics and other situational features that are difficult to change will reduce employees’ expectations for completing their work, reduce their work motivation, that is, their self-efficacy, and also hurt their affect (Jex and Gudanowski, 1992). Enterprise managers can also directly improve employees’ work motivation, that is, self-efficacy by organizing internal communication meetings or skill training. Only by managing the long-term motivation effect and the short-term affective strain can we effectively improve employees’ thriving at work.

### Limitations

Although the results of this study have significant academic and practical potential, there are still some areas to be improved. First, this study uses cross-sectional data; long-term data were not collected, which could affect the empirical results to a certain extent. Future research should include time-series data to supplement the lag effect between challenge and hindrance stressors and thriving at work.

Second, a single Chinese sample limits generalizability. The data source is mainly Chinese employees from the Pearl River Delta region of China. It is undeniable that Chinese employees sample has great research significance, since China has both extremely high economic growth and stressful work situations. Indeed, the sample of a single culture limits generalizability, therefore, we encourage scholars from different countries and cultural backgrounds to join us in cross-cultural sample research.

Third, this paper focuses on the two paths of affective strain and motivation. Each path only selects one representative variable. Therefore, in the study of affective path, only positive affect is selected, no negative affect. The role of negative affect and the counteracting effects of different affect are worthy of further study. We hope that both positive and negative affect will be incorporated into the model in the follow-up study to compare the mediating effects of positive and negative affect and test whether the challenge stressors may have a counteracting indirect effect on thriving through positive and negative affect.

Last, this paper does not analyze the boundary conditions of different types of stressors acting on thriving at work. At present, the controversy about the dichotomy model of stressors in positive psychology is that not all the empirical results of scholars can reflect the different effects of different types of stressors (McCarthy et al., 2019). For example, Prem et al. (2017) conducted an experimental study on the two dimensions of “vitality” and “learning” of challenge stressors and thriving at work. The results show that these two challenge stressors have a significant impact on learning, but do not significantly impact vitality. To a large extent, this is due to insufficient consideration of the regulatory variables affecting the interaction between the two stressors. The generation of stress is the result of the interaction between the individual and the environment. This means that the personal characteristics of the research object (such as initiative personality) and some external situational variables (such as leadership support and colleague trust) that affect the perception and evaluation of stress may have an impact on the formation and consequences of stress. Therefore, future research should further clarify the boundary conditions of the effect of stressors.

## Data Availability Statement

The raw data supporting the conclusions of this article will be made available by the authors, without undue reservation, to any qualified researcher.

## Ethics Statement

The studies involving human participants were reviewed and approved by Ethics Committee of Hunan Normal University. Written informed consent for participation was not required for this study in accordance with the national legislation and the institutional requirements.

## Author Contributions

YY and XL jointly built the model, conceived the research ideas, discussed the results, and revised the manuscript. The contribution of YY was mainly in theory, and the article was critically revised in the later period. XL’s contributions are mainly in methodological issues and data analysis. YY and XL were jointly responsible for the review of the article, ensuring that the research conducts the investigation in an appropriate manner to ensure the accuracy and completeness of the research. Both authors contributed to the article and approved the submitted version.

## Conflict of Interest

The authors declare that the research was conducted in the absence of any commercial or financial relationships that could be construed as a potential conflict of interest.
